# Patient's knowledge, attitudes, and practices toward acute coronary syndrome: a cross-sectional study

**DOI:** 10.3389/fcvm.2026.1675379

**Published:** 2026-03-09

**Authors:** Fei Wang, Jingping Wang, Rijun Wang, Jing Wang, Ruihua Zhao

**Affiliations:** 1 Department of Cardiovascular Medicine, Shanxi Cardiovascular Hospital, Taiyuan, China; 2 Department of Cardiovascular Medicine, Shanxi Key Laboratory of Heart Failure Precision Medicine, Taiyuan, China

**Keywords:** acute coronary syndrome, attitude, cross-sectional studies, health behavior, knowledge

## Abstract

**Background:**

Acute coronary syndrome imposes a heavy burden on health systems and can impair physical function and quality of life. Understanding patients' knowledge, attitudes, and practices is essential for timely care seeking and effective secondary prevention.

**Aim:**

This study was conducted to investigate patients' knowledge, attitudes, and practices toward acute coronary syndrome.

**Methods:**

A cross-sectional study was conducted among patients with acute coronary syndrome at Shanxi Cardiovascular Hospital (Taiyuan, China) from November 2022 to April 2024. Data were collected using a cardiovascular expert–reviewed questionnaire assessing knowledge, attitudes, and practices. Spearman correlation and logistic regression were performed using SPSS 27.0, and structural equation modeling was conducted using AMOS 26.0. A two-sided *p*-value <0.05 was considered statistically significant.

**Results:**

Among 475 valid questionnaires (67.58% male), mean knowledge, attitude, and practice scores were 4.16 (standard deviation 1.73), 45.05 (standard deviation 5.72), and 24.65 (standard deviation 6.37), respectively. Knowledge, attitudes, and practices were positively correlated (Spearman *r* = 0.212–0.427; *p* < 0.001). Structural equation modeling showed direct effects of knowledge on attitudes (standardized *β* = 0.293; *p* = 0.007) and practices (*β* = 0.172; *p* = 0.021), and of attitudes on practices (*β* = 0.525; *p* = 0.007). Knowledge also had an indirect effect on practices (*β* = 0.154; *p* = 0.004).

**Conclusion:**

Patients with acute coronary syndrome had limited knowledge but generally positive attitudes and practices. Improving symptom recognition, emergency response, and adherence-focused education may support timely care seeking and better management.

## Introduction

Acute coronary syndrome (ACS) comprises a spectrum of clinical conditions caused by acute myocardial ischemia or infarction after an abrupt reduction in coronary blood flow (1). The underlying mechanism is typically atherosclerotic plaque disruption with thrombus formation, which can rapidly compromise perfusion and cause myocardial injury (1). ACS remains a major global health problem, and its epidemiology continues to translate into a high burden of death and disability across regions (2). Clinical outcomes are highly time-sensitive, so patients’ recognition of warning signs and their immediate decisions during symptom onset are central to reducing prehospital delay (3). Atypical presentations and symptom ambiguity can complicate early self-assessment, which makes timely help seeking less likely when patients are uncertain about what they are experiencing (3). After the acute phase, secondary prevention becomes the dominant goal, and recommended management includes long-term pharmacotherapy, risk-factor control, and structured follow-up (4). Participation in cardiac rehabilitation and sustained adherence to prescribed regimens are part of this management pathway, yet patients' engagement can vary substantially in real-world settings (4, 5). Education in ACS therefore needs to address both the acute decision window and the longer-term behaviors that influence recurrent events and functional recovery (4, 5). Understanding how patients interpret symptoms, weigh perceived risk, and translate information into action is essential for planning education that is realistic for daily life (4, 6).

Knowledge–attitude–practice (KAP) surveys provide a practical framework to describe what people know, how they perceive a condition, and how they behave in relation to recommended care (6). Within this framework, knowledge relates to recognition and understanding of key information, attitudes reflect perceived seriousness and confidence, and practices capture intended or habitual actions aligned with prevention and response (6). In ACS and related high-risk populations, gaps in symptom recognition and uncertainty about appropriate first actions have been reported across settings, indicating persistent patient-level barriers to timely care (7–9). Studies among patients with ACS in Ethiopia reported incomplete recognition of warning signs and preferences that could delay emergency medical service use (7, 8). Research in Guyana among patients with type 2 diabetes similarly reported limitations in knowledge and beliefs related to ACS, including uncertainty about what to do when symptoms occur (7, 8). Evidence from Iran also described a sizable proportion of respondents who did not recognize the need for immediate hospitalization during ACS events, which is directly relevant to delay reduction (9). Beyond knowledge, attitudes can remain suboptimal, including low confidence in recognizing ACS or in deciding on emergency actions, even when some symptom knowledge is present (10). These findings matter because the acute phase requires rapid, decisive behavior, while secondary prevention requires sustained adherence and engagement over time (4, 5). Optimal post-ACS management depends on evidence-based therapy and follow-up, and adherence and rehabilitation participation shape longer-term outcomes that patients experience outside the hospital (4, 5). KAP measurement can therefore support more targeted education by showing whether limitations are mainly informational, motivational, or behavioral in nature (6).

In China, available KAP research has often focused on specific subgroups, such as patients with non-obstructive coronary ischemia or those undergoing percutaneous coronary intervention, which may not represent the broader spectrum of ACS encountered in routine coronary care (11, 12). Findings from these studies suggest that knowledge and attitudes relate to self-management behaviors, but the strength and direction of these relationships may vary across clinical contexts and patient populations (11, 12). Many reports remain descriptive and do not formally test whether attitudes mediate the association between knowledge and practices, even though mediation is relevant when education aims to increase both readiness to act during symptoms and sustained secondary prevention behaviors (6, 13). Structural equation modeling provides an approach to evaluate direct and indirect pathways among KAP domains within a single analytic framework, which can clarify how knowledge may translate into practice through attitudinal processes (12, 13). A KAP-based structural equation model has been used to examine linked pathways in other health topics, supporting its feasibility for evaluating complex behavioral mechanisms (13). The objective of this study was to assess patients' knowledge, attitudes, and practices toward ACS and to examine their interrelationships using correlation analysis and structural equation modeling (6, 12). We hypothesized that greater knowledge would be associated with more positive attitudes toward ACS (6, 12). We further hypothesized that more positive attitudes would be associated with more proactive practices (6, 12). Finally, we hypothesized that knowledge would be associated with practices both directly and indirectly through attitudes (12, 13).

## Methods

### Study design, setting and participants

This cross-sectional study was conducted among ACS patients from November, 2022, to April, 2024, at Shanxi Cardiovascular Hospital. Ethical approval was obtained from the Ethics Committee of Shanxi Cardiovascular Hospital, and informed consent was secured from all participants. Patients with medical history of ACS in the Electronic Medical Record were invited, including unstable angina, non-ST-segment elevation myocardial infarction, and ST-segment elevation myocardial infarction. Patients with communication barriers, unwillingness to communicate, severe myocarditis, pulmonary embolism, aortic dissection, cardiomyopathy, valvular disease/congenital heart disease, or other non-cardiac causes of chest pain were excluded.

### Ethical considerations

This study was conducted in accordance with the Declaration of Helsinki (2000) of the World Medical Association. Ethical approval was obtained from the Ethics Committee of Shanxi Cardiovascular Hospital (2022xxg050), and written informed consent was obtained from all participants.

### Data collection tools and measures

The questionnaire was developed with reference to relevant guidelines and literature and refined after item review by two cardiovascular experts to support content validity (6, 11, 12). A pilot study was conducted on 32 participants, and the internal consistency reliability was acceptable (Cronbach's *α* = 0.814).

Because the questionnaire was research-made, its content validity was assessed qualitatively through review by two cardiovascular experts for clinical relevance and wording correctness; formal content validity indices (CVI and CVR) were not calculated. The questionnaire did not include detailed modules on chronic use of potentially coronary risk–modifying medications (e.g., antidepressants, proton pump inhibitors, or nasal decongestants) or gastroesophageal reflux disease.

### Demographic questionnaire

Demographic information was collected using a structured questionnaire, including gender, age, marital status, cohabitant information, smoking history, and drinking history.

### Knowledge scale

The knowledge scale comprised 7 items (K1–K7). Each item was scored as 2 points for a correct answer and 0 points for an incorrect or “unsure” answer, with a total score ranging from 0 to 14; higher scores indicated better ACS-related knowledge.

### Attitude scale

The attitude scale included 11 items (A1–A11) rated on a five-point Likert scale. Items A1–A8 were scored from “Strongly agree” (5 points) to “Strongly disagree” (1 point), and items A9–A11 were reverse scored; total scores ranged from 11 to 55, with higher scores indicating more positive attitudes toward ACS.

### Practice scale

The practice scale contained 8 items (P1–P8). Items P1–P7 were rated on a five-point Likert scale from “Always” (5 points) to “Never” (1 point) for positive behaviors, and item P8 was a multiple-choice question addressing potential reasons for delayed care after ACS warning signs; total scores ranged from 7 to 35, with higher scores indicating more proactive practices.

### Scoring interpretation, validity and reliability

A score threshold of >70% of the total score in each dimension was used to define adequate knowledge, positive attitudes, and proactive practices (13). Content validity was supported through cardiovascular expert review and item refinement, and internal consistency reliability was acceptable in the pilot test (Cronbach's *α* = 0.814) (6, 11, 12).

### Data collection procedure and quality control

Questionnaires were distributed to ACS patients at the Coronary Heart Disease Care Group of Shanxi Cardiovascular Hospital through QR codes via WeChat. Researchers explained the study's purpose to subjects before the survey, and all subjects signed informed consent. Researchers were available to clarify any unclear questionnaire descriptions during the survey. Data collection ensured anonymity. Participants were informed that the survey was anonymous and would not affect their care, and they were encouraged to answer based on their actual behaviors. Research team members checked questionnaires for completeness, internal coherence, and reasonableness. Questionnaires with logical errors or uniform answers were deemed invalid and excluded.

### Sample size calculation

The calculation of sample size was based on the following formula employed in the cross-sectional study (14):

$$n={\left(\frac{Z_{1-\frac{\alpha }{2}}}{\delta }\right)}^{2}\times \;p\times (1-p)$$

where *n* denotes the sample size. Besides, *p*-value was assumed to be 0.5 to achieve the maximum sample size. *α* refers to the type I error, which was set to 0.05 in this case. Subsequently, $Z_{1-\frac{\alpha }{2}}$ was yielded 1.96. δ represents the allowable margin of error, which was set to 0.05, and at least 384 participants were required.

### Statistical and data analysis

SPSS 27.0 (IBM Corp., Armonk, NY, USA) and AMOS 26.0 (IBM Corp., Armonk, NY, USA) were used for analysis. Continuous variables were expressed as mean ± standard deviation, and categorical variables as *n* (%). Statistical tests included the Kolmogorov–Smirnov test (normality), Student's *t*-test (two-group comparison), one-way ANOVA (multi-group comparison), Mann–Whitney *U*-test (two-group comparison for skewed data), Kruskal–Wallis test (multi-group comparison for skewed data), Spearman correlation (association between KAP scores), and univariable/multivariable logistic regression (factors associated with dichotomized practice).[Table/Results] Structural equation modeling was conducted in AMOS, and model fit was evaluated using CMIN/DF, RMSEA, IFI, SRMR, TLI, and CFI. All tests were two-sided, and a *P*-value <0.05 was considered statistically significant.

## Results

### Baseline characteristics

A total of 509 questionnaires were collected. Excluding 7 responses completed in less than 60 s and 27 with all “unsure” answers in the knowledge section, 475 valid responses remained. Among these, 67.58% were male, and 52.21% were over 50 years old. The demographic variables collected included gender, age, marital status, cohabitant information, smoking history, and drinking history (Table 1). Most subjects were married (88.21%), living with partners (76.63%), non-smokers (64.00%), and non-drinkers (72.42%) (Table 1). Patients' knowledge, attitude, and practice scores regarding ACS were 4.16 ± 1.73, 45.05 ± 5.72, and 24.65 ± 6.37, respectively (Table 1).

**Table 1:** Basic information of participants and KAP score (*N* = 475).

Variables	*N* (%)	Knowledge (mean ± SD)	*P*	Attitude (mean ± SD)	*P*	Practice (mean ± SD)	*P*
Total score		4.16 ± 1.73		45.05 ± 5.72		24.65 ± 6.37	
Gender			0.201		0.086		0.917
Male	321 (67.58)	4.09 ± 1.74		44.75 ± 5.68		24.71 ± 6.08	
Female	154 (32.42)	4.32 ± 1.71		45.66 ± 5.78		24.54 ± 6.95	
Age (years)			0.941		0.007		0.008
<30	40 (8.42)	4.20 ± 1.90		44.58 ± 5.92		21.13 ± 7.57	
31–40	89 (18.74)	4.28 ± 1.63		46.84 ± 5.18		25.84 ± 6.87	
41–50	98 (20.63)	4.10 ± 1.75		45.20 ± 5.48		24.99 ± 6.41	
>50	248 (52.21)	4.14 ± 1.74		44.42 ± 5.86		24.67 ± 5.77	
Marital status			0.315		0.980		0.122
Unmarried	46 (9.68)	4.22 ± 1.88		45.35 ± 5.54		22.89 ± 7.85	
Married	419 (88.21)	4.18 ± 1.70		45.03 ± 5.65		24.92 ± 6.13	
Divorced/widowed	10 (2.11)	3.30 ± 2.11		44.40 ± 9.38		21.80 ± 7.36	
Cohabitant information			0.097		0.259		0.018
Living alone	36 (7.58)	3.58 ± 1.81		43.86 ± 6.02		22.94 ± 7.79	
Partner	364 (76.63)	4.27 ± 1.68		45.29 ± 5.63		25.17 ± 6.10	
Children	56 (11.79)	3.91 ± 2.00		44.64 ± 6.17		23.50 ± 6.24	
Other	19 (4.00)	4.00 ± 1.49		43.79 ± 5.41		21.37 ± 7.41	
Smoking			0.001		0.347		0.016
Yes	171 (36.00)	3.82 ± 1.74		44.73 ± 5.93		23.92 ± 5.70	
No	304 (64.00)	4.35 ± 1.70		45.23 ± 5.60		25.07 ± 6.69	
Drinking			0.008		0.507		0.239
Yes	131 (27.58)	3.79 ± 1.80		44.85 ± 5.83		24.22 ± 6.16	
No	344 (72.42)	4.30 ± 1.68		45.12 ± 5.68		24.82 ± 6.45	

*P*-values were calculated using independent-samples *t*-test (two groups) or one-way ANOVA (more than two groups) for normally distributed data, and Mann–Whitney *U*-test (two groups) or Kruskal–Wallis test (more than two groups) for non-normally distributed data, as appropriate.

### KAP distribution

Table 2 presents the item-level correct response rates for the knowledge dimension. Accuracy rates in the knowledge dimension ranged from 26.11% to 84.63%. The lowest accuracy (26.11%) was for recognizing that morphine can be appropriately given for pain relief in severe cases (K6). Only 49.68% accurately acknowledged that lowering blood lipids can prevent the risk of ACS, but is not of high priority (K5). Additionally, nearly a half (54.11%) correctly identified pain or discomfort in the jaw, neck, or back as common ACS warning signs (Table 2).

**Table 2:** Knowledge dimension of the participants.

Knowledge	Correct rate (%)
1. Aspirin is a commonly used antiplatelet medication.	309 (65.05)
2. Chest pain or discomfort is a common warning sign of acute coronary syndrome.	402 (84.63)
3. Pain or discomfort in the arm or shoulder is a common warning sign of acute coronary syndrome.	279 (58.74)
4. Pain or discomfort in the jaw, neck, or back is a common warning sign of acute coronary syndrome.	257 (54.11)
5. Lowering blood lipids (such as total cholesterol) is not very significant in preventing acute coronary syndrome.	236 (49.68)
6. Morphine can be appropriately given for pain relief in severe cases.	124 (26.11)
7. Measures such as quitting smoking and drinking are not very significant in preventing acute coronary syndrome.	370 (77.89)

Values are presented as *n* (%); no hypothesis testing was performed for these descriptive items.

Table 3 summarizes participants' responses to each attitude item on the five-point Likert scale. Positivity rates in the attitudes dimension varied from 56.84% to 95.16%. The lowest positivity rate (56.84%) was for confidence in performing emergency self-rescue or correctly assisting others during an acute coronary syndrome event (A5). Only 57.26% expressed confidence in differentiating ACS symptoms from other related disease symptoms (A4) (Table 3). Moreover, the limited proportion (69.68%) held positive attitudes towards the recognition of the signs and symptoms when an ACS attack occurs.

**Table 3:** Attitude dimension of the participants.

Attitude	*N* (%)				
	Strongly agree	Agree	Neutral	Disagree	Strongly disagree
1. You are willing to actively learn about the warning signs of acute coronary syndrome.	304 (64)	145 (30.53)	23 (4.84)	1 (0.21)	2 (0.42)
2. You are willing to actively learn about the emergency handling methods for acute coronary syndrome warning signs.	314 (66.11)	138 (29.05)	19 (4)	1 (0.21)	3 (0.63)
3. You believe you have the confidence to recognize the signs and symptoms of an acute coronary syndrome attack in yourself or others.	194 (40.84)	137 (28.84)	111 (23.37)	31 (6.53)	2 (0.42)
4. You believe you have the confidence to differentiate acute coronary syndrome symptoms from other possible related disease symptoms.	155 (32.63)	117 (24.63)	131 (27.58)	61 (12.84)	11 (2.32)
5. When an acute coronary syndrome occurs in yourself or others, you believe you have the confidence to perform emergency self-rescue or correctly assist others.	150 (31.58)	120 (25.26)	129 (27.16)	62 (13.05)	14 (2.95)
6. You believe you can follow medical advice to undertake necessary preventive measures for acute coronary syndrome.	225 (47.37)	187 (39.37)	50 (10.53)	7 (1.47)	6 (1.26)
7. If chest pain persists for 15 min without relief, you will call 120.	283 (59.58)	152 (32)	24 (5.05)	13 (2.74)	3 (0.63)
8. If you feel you are having an attack, you will choose to go to the nearest hospital.	272 (57.26)	156 (32.84)	23 (4.84)	21 (4.42)	3 (0.63)
9. If you feel you are about to have an attack, you would rather wait for someone to drive me to the hospital than call an ambulance.	48 (10.11)	40 (8.42)	29 (6.11)	222 (46.74)	136 (28.63)
10. Because of the high expense of hospitalization, you will absolutely make sure you about to have an attack before going to the hospital.	43 (9.05)	54 (11.37)	47 (9.89)	223 (46.95)	108 (22.74)
11. You feel that emergency medical services respond too slowly/that emergency medical services are not as good as self-medication.	27 (5.68)	34 (7.16)	50 (10.53)	236 (49.68)	128 (26.95)

Values are presented as *n* (%); no hypothesis testing was performed for these descriptive items.

Table 4 shows the response distribution for each practice item and the reported reasons for delayed care seeking after warning signs. Practice adherence rates ranged from 29.90% to 87.79%. The least proportion (29.90%) actively attended lectures and training related to ACS and its warning signs (P2), and only 33.47% actively learned about these topics (P1). When surrounding persons collapse and lose consciousness, merely 33.69% can promptly perform CPR (P4). Regarding delays in seeking medical attention after noticing warning signs, 52.21% indicated that previously noticed warning signs disappeared, followed by not being well-informed about the symptoms (50.53%), and feeling that they could control the symptoms and they were not severe enough to warrant a hospital visit (38.74%) (Table 4).

**Table 4:** Practice dimension of the participants.

Practice	*N* (%)				
	Always	Often	Sometimes	Rarely	Never
1. The frequency with which you actively learn about acute coronary syndrome and its warning signs.	80 (16.84)	79 (16.63)	104 (21.89)	145 (30.53)	67 (14.11)
2. The frequency with which you attend lectures and training related to acute coronary syndrome and its warning signs.	72 (15.16)	70 (14.74)	92 (19.37)	131 (27.58)	110 (23.16)
3. You keep emergency medications such as aspirin, nitroglycerin, and quick-acting heart-relief pills at home.	187 (39.37)	108 (22.74)	49 (10.32)	42 (8.84)	89 (18.74)
4. When someone around you collapses and loses consciousness, you will promptly perform CPR.	111 (23.37)	49 (10.32)	71 (14.95)	83 (17.47)	161 (33.89)
5. You strictly follow the doctor's guidance in taking medication.	324 (68.21)	93 (19.58)	29 (6.11)	18 (3.79)	11 (2.32)
6. To prevent acute coronary syndrome, you have proactively changed your lifestyle/diet.	246 (51.79)	114 (24)	69 (14.53)	28 (5.89)	18 (3.79)
7. To prevent acute coronary syndrome, you have become more focused on exercise and weight management.	251 (52.84)	111 (23.37)	61 (12.84)	33 (6.95)	19 (4)
8. What do you find troubling about seeking timely medical attention after noticing warning signs of acute coronary syndrome? (Multiple choices)					
The previously noticed warning signs disappeared.	248 (52.21)				
I am not well-informed about the warning sign symptoms.	240 (50.53)				
The symptoms occur outside of regular daily life or work hours.	104 (21.89)				
The symptoms occur in a public place.	82 (17.26)				
I feel I can control the symptoms and they are not severe enough to warrant a hospital visit.	184 (38.74)				
I feel embarrassed to ask for help.	60 (12.63)				
I do not want to trouble others.	100 (21.05)				
I feel fearful about what might happen.	104 (21.89)				
I feel fearful about going to the hospital.	86 (18.11)				
Other	68 (14.32)				

Values are presented as *n* (%); no hypothesis testing was performed for these descriptive items.

### Spearman correlation analysis and logistic regression

Table 5 reports the univariable and multivariable logistic regression results for factors associated with proactive practices. Spearman correlation analysis demonstrated positive correlations between knowledge and attitude (*r* = 0.212, *P* < 0.001), knowledge and practice (*r* = 0.283, *P* < 0.001), and attitude and practice (*r* = 0.427, *P* < 0.001) (Supplementary Table S1). A cut-off value of 24.5 was used to categorize practice scores, with 250 participants exceeding this threshold. Univariable analysis revealed an inverse association between smoking and practice (OR = 0.644, 95% CI: 0.442–0.938, *P* = 0.022). Multivariable analysis identified significant positive associations of knowledge (OR = 1.411, 95% CI: 1.243–1.602, *P* < 0.001), attitude (OR = 1.143, 95% CI: 1.098–1.190, *P* < 0.001), and age groups of 41–50 years (OR = 2.905, 95% CI: 1.223–6.900, *P* = 0.016) and over 50 years (OR = 3.252, 95% CI: 1.470–7.195, *P* = 0.004) with practice (Table 5).

**Table 5:** Univariable and multivariable logistic regression of practice scores.

Characteristics	Univariable logistic regression		Multivariable logistic regression	
	OR (95% CI)	*P*	OR (95% CI)	*P*
Knowledge	1.483 (1.319–1.667)	<0.001	1.411 (1.243–1.602)	<0.001
Attitude	1.148 (1.106–1.192)	<0.001	1.143 (1.098–1.190)	<0.001
Gender				
Male	1.041 (0.709–1.530)	0.836		
Female	ref			
Age				
Under 30 years	ref		ref	
31–40 years	2.078 (0.961–4.496)	0.063	1.916 (0.808–4.543)	0.140
41–50 years	2.187 (1.021–4.684)	0.044	2.905 (1.223–6.900)	0.016
Over 50 years	2.255 (1.124–4.524)	0.022	3.252 (1.470–7.195)	0.004
Marital status				
Single	ref			
Married	1.381 (0.749–2.544)	0.301		
Other	0.794 (0.197–3.192)	0.745		
Cohabitant information				
Living alone	ref			
Partner	1.746 (0.872–3.495)	0.116		
Children	1.129 (0.484–2.632)	0.779		
Other	1.018 (0.330–3.140)	0.975		
Smoking				
Yes	0.644 (0.442–0.938)	0.022	0.668 (0.435–1.025)	0.065
No	ref		ref	
Drinking				
Yes	0.778 (0.520–1.164)	0.222		
No	ref			

*P*-values were obtained from univariable and multivariable logistic regression models; odds ratios (ORs) are reported with 95% confidence intervals (CIs).

### SEM analysis

**Figure 1:**
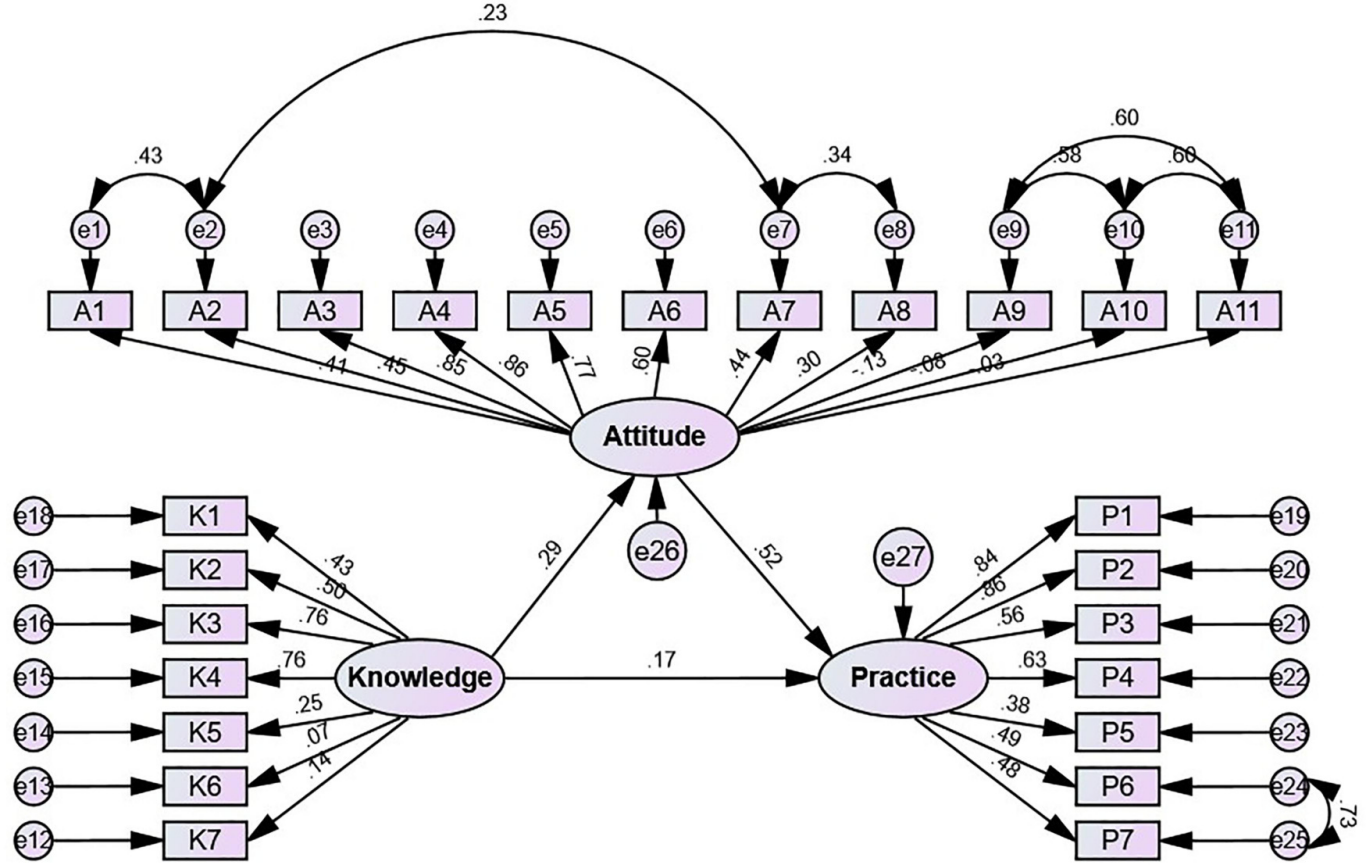
Structural equation model showing the associations between KAP scores. All variables are observed variables. Direction of causality is indicated by single-headed arrows. The standardized path coefficients are presented alongside the arrows.

Figure 1 and Table 6 present the structural equation model and the standardized direct and indirect effects, and model fit indices are provided in Supplementary Table S2. The goodness-of-fit indices indicated a satisfactory model fit for the SEM (CMIN/DF = 3.488, RMSEA = 0.072, IFI = 0.852, SRMR = 0, TLI = 1, CFI = 1) (Supplementary Table S2). The SEM suggests that the knowledge has direct impacts on both attitude (*β* = 0.293, 95%CI: 0.163–0.415, *P* = 0.007) and practice (*β* = 0.172, 95% CI: 0.023–0.280, *P* = 0.021), and the attitude has a direct impact on practice (*β* = 0.525, 95% CI: 0.431–0.614, *P* = 0.007). Additionally, the knowledge also has an indirect impact on practice (*β* = 0.154, 95% CI: 0.097–0.233, *P* = 0.004) (Figure 1 and Table 6).

**Table 6:** Direct and indirect effects of KAP scores from structural equation model.

Model paths	Standardized direct effects	95% CI	*P*	Standardized indirect effects	95% CI	*P*
Knowledge→Attitude	0.293	0.163–0.415	0.007			
Knowledge→Practice	0.172	0.023–0.280	0.021	0.154	0.097–0.233	0.004
Attitude→Practice	0.525	0.431–0.614	0.007			

Standardized direct and indirect effects were estimated using structural equation modeling (AMOS 26.0); *P*-values and 95% confidence intervals (CI) are reported for each path.

## Discussion

This study assessed patients' knowledge, attitudes, and practices toward acute coronary syndrome and examined how these domains relate to each other using correlation analysis and structural equation modeling. We found limited knowledge alongside generally positive attitudes and practices. The weakest areas clustered around atypical symptom recognition, confidence in emergency response, and engagement in learning activities. The SEM results suggested that knowledge related to practice both directly and through attitudes, and attitudes showed the strongest link with practice.

Prior studies have also reported gaps in ACS knowledge. In Bangladesh, many patients showed normal to poor knowledge (14). In Ethiopia, patients often missed atypical symptoms and many preferred self-transport rather than emergency medical services (6). In Iran, a substantial proportion did not recognize the need for immediate hospitalization during ACS events (8). In our sample, recognition of jaw, neck, or back discomfort as warning signs was limited, and misconceptions around acute management were common. The low correct response rate for morphine use for severe pain suggests uncertainty about emergency medications, despite morphine being discussed in prehospital pain management recommendations (15). Only about half of participants correctly interpreted the role of lipid-lowering therapy in relation to ACS, indicating that prevention concepts were not well understood.

Attitudes showed a clear weak point in perceived ability to act during an acute event. Many participants lacked confidence in emergency self-rescue or helping others, and CPR readiness was also low. These findings align with the idea that knowing symptoms does not automatically translate into feeling prepared to respond (16). Confidence in distinguishing ACS from other causes of chest discomfort was also limited, which is understandable given symptom overlap with noncardiac chest pain conditions (17). Such uncertainty can contribute to hesitation and delayed decisions when symptoms occur (18).

Practice patterns showed a similar gap between medication adherence and proactive learning. Most participants reported following medication guidance, yet participation in lectures or self-directed learning was low. Underuse of secondary prevention programs has been noted in cardiovascular populations (19), and our data suggest that access and motivation for education remain practical barriers. Delay reasons further pointed to misinterpretation of transient symptom relief: more than half reported waiting because symptoms disappeared. This is concerning because ACS symptoms can fluctuate, and transient improvement may still precede deterioration (20).

The pathway results provide a practical interpretation for education design. Knowledge related to attitudes, and attitudes related strongly to practice; this supports focusing on both information and patients' readiness to act, rather than information alone (21). Logistic regression also suggested that older age groups were more likely to show proactive practices, while smoking tended to relate to poorer practice, echoing the challenge of risk perception and behavior change in smokers (22).

Strengths include a sample covering unstable angina, NSTEMI, and STEMI and use of SEM to test direct and indirect pathways across KAP. Limitations include self-reported measures with social desirability bias, a cross-sectional design, recruitment from a single tertiary center with comorbid conditions excluded, use of a study-made questionnaire reviewed by two experts without formal CVI/CVR, and lack of data on chronic risk–modifying medications or GERD, which may limit external validity and causal inference.

## Conclusion

Among 475 ACS patients, knowledge was limited while attitudes and practices were generally positive. KAP scores were correlated. SEM showed knowledge influenced attitude and practice, with attitude mediating. Older age was linked to better practice.

To meet benchmarks for timely ACS care and secondary prevention, hospitals and local health authorities should standardize symptom-recognition and emergency-response education as part of admission and discharge workflows. Core messages should cover atypical warning signs, immediate EMS activation (calling 120), and adherence to prescribed therapy. Discharge plans should include referral to cardiac rehabilitation, simple follow-up channels (telephone/WeChat), and materials for family members. Community-level programs can expand CPR and basic life support training and reinforce when to seek urgent care, prioritizing groups with lower practice levels such as smokers and younger patients.

We suggest using a brief bedside checklist to teach patients and families key ACS warning signs, including jaw/neck/back discomfort, and to practice the decision to call 120. Provide short, repeated education (leaflets and WeChat messages) on medication adherence and lifestyle change, and offer targeted counseling for smokers. Encourage participation in cardiac rehabilitation and provide opportunities for CPR practice during hospitalization or early follow-up.

## Data Availability

The original contributions presented in the study are included in the article/Supplementary Material, further inquiries can be directed to the corresponding authors.
