# Supplementary material for: Patient's knowledge, attitudes, and practices toward acute coronary syndrome: a cross-sectional study
Source: Front Cardiovasc Med. 2026 Mar 9;13:1675379. doi: 10.3389/fcvm.2026.1675379 (PMC13006618; doi:10.3389/fcvm.2026.1675379)
Supplement: Supplementary file 1 [file Datasheet1.pdf]

Knowledge, Attitude, and Practice of Acute Coronary Syndrome Patients towards the Disease and Warning Signs

Part 1: Basic Information

**1.gender：**	a. male<br>b. female
**2.age：**	a. Under 30 years<br>b.31-40 years<br>c.41-50 years<br>d. over 50 years
**3.marital status：**	a. single<br>b. married<br>c. other
**4.cohabitant information：**	a. living alone<br>b. partner<br>c. children<br>d. Caregiver<br>e. other
**5.do you smoke：**	a. yes<br>b. no
**6.do you drink：**	a. yes<br>b. nos

Part 2: Knowledge of Acute Coronary Syndrome and Warning Signs

**1. Aspirin is a commonly used antiplatelet medication.**	a. yes	b. no	c. unsure
**2. Chest pain or discomfort is a common warning sign of acute coronary syndrome.**	a. yes	b. no	c. unsure
**3. Pain or discomfort in the arm or shoulder is a common warning sign of acute coronary syndrome.**	a. yes	b. no	c. unsure

**4. Pain or discomfort in the jaw, neck, or back is a common warning sign of acute coronary syndrome.**	a. yes	b. no	c. unsure
**5. Lowering blood lipids (such as total cholesterol) is not very significant in preventing acute coronary syndrome.**	a. yes	b. no	c. unsure
**6. Morphine can be appropriately given for pain relief in severe cases.**	a. yes	b. no	c. unsure
**7. Measures such as quitting smoking and drinking are not very significant in preventing acute coronary syndrome.**	a. yes	b. no	c. unsure

Part 3: Attitudes towards Acute Coronary Syndrome and Warning Signs

**1. You are willing to actively learn about the warning signs of acute coronary syndrome.**	a. strongly agree	b. agree	c. neutral	d. disagree	e. strongly disagree
**2. You are willing to actively learn about the emergency handling methods for acute coronary syndrome warning signs.**	a. strongly agree	b. agree	c. neutral	d. disagree	e. strongly disagree
**3. You believe you have the confidence to recognize the signs and symptoms of an acute coronary syndrome attack in yourself or others.**	a. strongly agree	b. agree	c. neutral	d. disagree	e. strongly disagree
**4. You believe you have the confidence to differentiate acute coronary syndrome symptoms from other possible related disease symptoms.**	a. strongly agree	b. agree	c. neutral	d. disagree	e. strongly disagree
**5. When an acute coronary syndrome occurs in yourself or others, you believe you have the confidence to perform emergency self-rescue or correctly assist others.**	a. strongly agree	b. agree	c. neutral	d. disagree	e. strongly disagree
**6. You believe you can follow medical advice to undertake necessary preventive measures for acute coronary syndrome.**	a. strongly agree	b. agree	c. neutral	d. disagree	e. strongly disagree
**7. If chest pain persists for 15 minutes without relief, I will call 120.**	a. strongly agree	b. agree	c. neutral	d. disagree	e. strongly disagree
**8. If I feel I am having an attack, I will choose to go to the**	a. strongly	b. agree	c. neutral	d. disagree	e. strongly

**nearest hospital.**	agree				disagree
**9. If I feel I am about to have an attack, I would rather wait for someone to drive me to the hospital than call an ambulance.**	a. strongly agree	b. agree	c. neutral	d. disagree	e. strongly disagree
**10. Because going to the hospital costs money, I will absolutely make sure I am about to have an attack before going to the hospital.**	a. strongly agree	b. agree	c. neutral	d. disagree	e. strongly disagree
**11. You feel that emergency medical services respond too slowly/that emergency medical services are not as good as self-medication.**	a. strongly agree	b. agree	c. neutral	d. disagree	e. strongly disagree

Part 4: Practices in Acute Coronary Syndrome Warning Signs

Regarding the options for "Frequency": Always: Practiced more than 9 times in the past 2 months, Often: Practiced 7 to 9 times in the past 2 months, Sometimes: Practiced 4 to 6 times in the past 2 months, Rarely: Practiced 1 to 3 times in the past 2 months, Never: Practiced 0 times in the past 2 months

**1. The frequency with which you actively learn about acute coronary syndrome and its warning signs.**	a. always	b. often	c. sometimes	d. rarely	e. never
**2. The frequency with which you attend lectures and training related to acute coronary syndrome and its warning signs.**	a. always	b. often	c. sometimes	d. rarely	e. never
**3. You keep emergency medications such as aspirin, nitroglycerin, and quick-acting heart-relief pills at home.**	a. always	b. often	c. sometimes	d. rarely	e. never
**4. When someone around you collapses and loses consciousness, you will promptly perform CPR.**	a. always	b. often	c. sometim	d. rarely	e. never

			es		
**5. You strictly follow the doctor's guidance in taking medication.**	a. always	b. often	c. sometimes	d. rarely	e. never
**6. To prevent acute coronary syndrome, you have proactively changed your lifestyle/diet.**	a. always	b. often	c. sometimes	d. rarely	e. never
**7. To prevent acute coronary syndrome, you have become more focused on exercise and weight management.**	a. always	b. often	c. sometimes	d. rarely	e. never
**8. What do you find troubling about seeking timely medical attention after noticing warning signs of acute coronary syndrome? (Multiple choices)**	a. The previously noticed warning signs disappeared	b. I am not well-informed about the warning sign symptoms.	c. The symptoms occur outside of regular daily life or work hours	d. The symptoms occur in a public place	e. I feel I can control the symptoms and they are not severe enough to warrant a hospital visit.
	f. I feel embarrassed to ask for help	g. I do not want to trouble others	h. I feel fearful about what might happen	i. I feel fearful about going to the hospital	j. Other
