# Supplementary material for: Patient's knowledge, attitudes, and practices toward acute coronary syndrome: a cross-sectional study
Source: Front Cardiovasc Med. 2026 Mar 9;13:1675379. doi: 10.3389/fcvm.2026.1675379 (PMC13006618; doi:10.3389/fcvm.2026.1675379)
Supplement: Supplementary file 2 [file Datasheet2.docx]

**Table S1. Spearman’s correlation analysis of KAP scores**

|  | Knowledge | Attitude | Practice |
| --- | --- | --- | --- |
| Knowledge | 1 |  |  |
| Attitude | 0.212 (P<0.001) | 1 |  |
| Practice | 0.283 (P<0.001) | 0.427 (P<0.001) | 1 |

# Table S2. Goodness-of-fit for structural equation model

| **Model Fit Indices** | **Reference** | **Measured results** |
| --- | --- | --- |
| CMIN/DF | 1-3 excellent，3-5 good | 3.488 |
| RMSEA | <0.08 good | 0.072 |
| IFI | >0.8 good | 0.852 |
| SRMR | <0.08 Good | 0.000 |
| TLI | >0.8 Good | 1.000 |
| CFI | >0.8 Good | 1.000 |

Abbreviations: CMIN/DF: Chi-Square Minimum Discrepancy divided by Degrees of Freedom; RMSEA: Root Mean Square Error of Approximation; IFI: Incremental Fit Index; SRMR: Standardized Root Mean Square Residual; TLI: Tucker-Lewis Index (also known as the Non-Normed Fit Index, NNFI); CFI: Comparative Fit Index
